# Impact of rubidium imaging availability on management of patients with acute chest pain

**DOI:** 10.1007/s12350-022-02923-8

**Published:** 2022-02-23

**Authors:** Akasha Shaukat Ali, Vincent Finnerty, Francois Harel, Guillaume Marquis-Gravel, Alain Vadeboncoeur, Matthieu Pelletier-Galarneau

**Affiliations:** 1 Department of Medical Imaging, Montreal Heart Institute, Montreal, QC H1T 1C8 Canada; 2 Department of Medicine, Montreal Heart Institute, Montreal, QC Canada; 3 Emergency Department, Montreal Heart Institute, Montreal, QC Canada

**Keywords:** Chest pain, Positron emission tomography, Rubidium-82, Rb-82, Myocardial perfusion imaging, dolor torácico, tomografía de emisión de positrones, rubidio-82, Rb-82, imágenes de perfusión miocárdica

## Abstract

**Objective:**

Evaluate the impact of 82-Rubidium positron emission tomography (PET) myocardial perfusion imaging (MPI) availability on patient management presenting at the emergency department (ED) with chest pain (CP).

**Methods:**

This is a single-center retrospective study of clinical databases. Patients presenting with CP with a non-definitive suspicion of acute coronary syndrome (ACS) at the ED between April 2016 and February 2020 were divided into 2 groups based on PET availability. The proportion of invasive coronary angiography (ICA) without significant coronary artery disease (CAD), length of stay (LoS), and additional downstream testing were evaluated.

**Results:**

There were 21,242 ED visits for CP without definitive ACS: 5,492 when PET is not available and 15,750 when PET is available. When PET is available, proportion of patients undergoing a MPI study was greater (20.7% vs 17.6%, *P*<0.0001), proportion of ICA without significant CAD was similar (18.5% vs 21.4%, *P*=0.24), and median ED LoS was shorter (16.6 vs 18.1 hours, *P*=0.03). Patients undergoing SPECT MPI had significantly more downstream testing (8.9% vs 6.4%, *P*=0.003) and a higher rate of coronary angiogram without significant CAD (21.2% vs 14.2%, *P*=0.09) compared to those who underwent PET MPI.

**Conclusion:**

Availability of PET MPI was associated with an increased number of MPI referral from the ED, similar rates of ICA without significant CAD, decreased LoS, and fewer downstream testing.

**Supplementary Information:**

The online version contains supplementary material available at 10.1007/s12350-022-02923-8.

## Spanish Abstract

**Objetivo:**

Evaluar el impacto de la tomografía por emisión de positrones (PET) con 82-Rubidio y la disponibilidad de imágenes de perfusión miocárdica (MPI) en el manejo de los pacientes que se presentan en el servicio de urgencias (ED) con dolor torácico (CP).

**Métodos:**

Este es un estudio retrospectivo de bases de datos clínicas de un solo centro. Pacientes que presentaron CP con sospecha no definitiva de síndrome coronario agudo (ACS) en el ED entre abril de 2016 y febrero de 2020, se dividieron en 2 grupos según la disponibilidad de PET. Se evaluó la proporción de angiografía coronaria invasiva (ICA) sin enfermedad arterial coronaria (CAD) significativa, la duración de la estancia (LoS) y las pruebas posteriores adicionales.

**Resultados:**

Hubo 21,242 visitas al ED por CP sin ACS definitivo: 5,492 cuando no se dispone de PET y 15.750 cuando se dispone de PET. Cuando se dispone de PET, la proporción de pacientes sometidos a estudio de MPI fue mayor (20.7% vs 17.6%, p=0.03). Los pacientes que se sometieron a SPECT MPI tuvieron significativamente más pruebas posteriores (8.9 % frente a 6.4 %, p = 0.003) y una tasa más alta de angiografía coronaria sin CAD significativa (21.2 % frente a 14.2 %, p = 0.09) en comparación con los que se sometieron a PET MPI.

**Conclusión:**

La disponibilidad de PET MPI se asoció con un mayor número de referencias de MPI desde el ED, tasas similares de ICA sin CAD significativa, disminución de LoS y menos pruebas posteriores.

## Introduction

Chest pain (CP) and related symptoms are among the most frequent complaints of patients presenting to emergency departments (ED).^1^ Management of patients with CP but without a clear diagnosis of ACS represents a diagnostic challenge for physicians and may contribute to downstream costs and potential risk if unnecessary invasive testing is performed. For example, unnecessary admissions lead to high health care costs and excessive resource utilization. Conversely, patients who are inappropriately discharged face delay in treatment which is associated with increased morbidity and mortality.^2–5^ Moreover, management of CP in ED represents a major source of medical-legal actions against physicians.^6^ In that setting, patients presenting with CP at ED without clear evidence of ACS may require comprehensive cardiac evaluation for diagnosis and risk stratification.^7^

The Montreal Heart Institute (MHI) is a quaternary care facility dedicated to the treatment of patients with cardiac diseases. As an extension of MHI’s mission, the MHI ED is also dedicated to cardiac diseases, with CP being one of the most common reason for emergency room consultation. Following current appropriate use criteria (AUC), patients presenting with symptoms compatible with ischemic heart disease but without definite evidence of ACS are often referred to the Nuclear Cardiology lab for a myocardial perfusion imaging (MPI) study.^8^ For intermediate-risk patients, imaging is typically performed prior to discharge while for lower-risk patients, imaging may be deferred to after discharge and performed on an outpatient basis with a specific appointment. In January 2017, positron emission tomography (PET) MPI with the radiotracer Rubidium-82 Chloride was introduced at MHI Nuclear Cardiology lab. At MHI, when PET MPI is available, it is the modality of choice over single photon emission computed tomography (SPECT) MPI to image patients referred from the ED. Indeed, PET MPI presents several advantages over SPECT MPI for the evaluation of CP in the ED settings, such as cardiac PET’s improved diagnostic accuracy, lower patient and health care worker radiation dose, ability to quantify myocardial blood flow, and shorter imaging time.^9^ With Rubidium-82, complete rest-stress imaging can routinely be accomplished within 30-45 minutes. However, from November 2018 to February 2019, all patients referred for a MPI study were imaged with SPECT due to the unavailability of Rubidium-82 generator. The purpose of this study is to evaluate the impact of PET availability on the management of patients presenting with CP but inconclusive diagnosis of ACS at the ED. The pre-defined outcomes were as follows: the proportion of patients undergoing angiography, the proportion of angiography without significant epicardial stenosis, ED length of stay (LoS), proportion of patients undergoing MPI vs other tests, and downstream additional testing.

## Materials and Methods

### Patient Population

This retrospective study was approved by our institutional review board which determined that the requirement to obtain informed consent was waived because there was no patient involvement, change in their management or treatment, and no patient risk. In addition to patient chart reviews, data from three clinical databases were merged for the analysis by a trained investigator: these included the ED database, the nuclear medicine database, and the coronary angiography database. Patients presenting with CP to the ED were identified using the “complaint code” from the ED database, which represents the chief complaint at triage presentation, with patients categorized as “anginal chest pain” and “non-anginal chest pain” as per the Canadian Triage and Acuity Scale (CTAS) included in the data analysis. Initial evaluation of patients with CP at the ED includes assessment of symptoms, medical history, previous tests results, risk factors, ECG, and high-sensitivity troponins levels and kinetics. Patients presenting with angina equivalents (e.g., dyspnea) were excluded. Patients presenting with STEMI and NSTEMI were identified using discharge codes (ICD-10 diagnostic codes I21.x) and were excluded from the analysis.

### Study Periods and Groups

**Figure 1:**
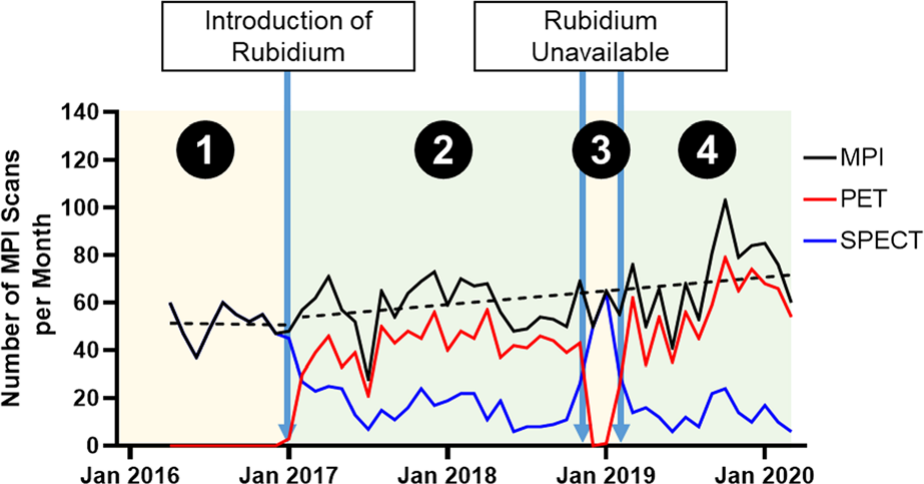
Number of positron emission tomography (PET) myocardial perfusion imaging (MPI) study (red) and total number of MPI (black) study referred from the ED for patients presenting with chest pain. Dotted black line represents the linear regression of the total MPI number before and after January 2017. The 4 Periods of the study are presented: the year preceding the introduction of Rubidium-82 from Apr 2016 to Dec 2016 (Period 1), the period when Rubidium-82 was available from Jan 2017 to Oct 2018 (Period 2), the period when Rubidium-82 was unavailable from Nov 2018 to Feb 2019 (Period 3), and the year following when Rubidium-82 resumed availability from Mar 2019 to Feb 2020 (Period 4)

Four time periods were defined based on Rubidium-82 availability (Figure 1): the year preceding the introduction of Rubidium-82 from Apr 2016 to Dec 2016 (Period 1), the period when Rubidium-82 was available from Jan 2017 to Oct 2018 (Period 2), the period when Rubidium-82 was unavailable from Nov 2018 to Feb 2019 (Period 3), and the year following when Rubidium-82 resumed availability from Mar 2019 to Feb 2020 (Period 4). Period 1 start date corresponds to the creation of the ED database. The Period 4 end date was chosen to exclude the COVID-19 pandemic. For the purpose of the analysis, patients were divided into 2 groups based on Rubidium-82 availability: PET Available (Period 2 and 4) and PET Not Available (Period 1 and 3).

### Myocardial Perfusion Imaging

When PET was not available, all MPI studies were performed with SPECT. When PET was available, it was the favored MPI modality for inpatient imaging, although occasionally inpatients were imaged with SPECT when the number of requisitions exceeded PET capacity. All exercise MPI studies were performed with SPECT. For outpatient imaging, each requisition was protocol to PET or SPECT by a physician. Generally, PET is the preferred modality with SPECT reserved for lower-risk non-obese patients. Patients with breast implants, history of by-pass surgery, prior positive PET MPI study, or when flow quantification was judged necessary (e.g., intermediate lesions on invasive coronary angiography [ICA]) were systematically imaged with PET. All SPECT imaging was performed on Siemens Symbia T6 dual-head SPECT/CT hybrid scanner (Siemens Medical Solution, USA) with cardiofocal collimators (IQ-SPECT) and interpretation was conducted using both images with and without CT attenuation correction. Between 500 and 1100 MBq of ^99m^Tc-tetrofosmin were injected intravenously for rest and stress imaging, and either pharmacological stress with dipyridamole (0.142 mg/kg/min over 4 minutes) or exercise treadmill stress was performed. Single-day rest-stress imaging was favored for ED patients whenever possible. PET imaging was performed on Siemens Biograph mCT Flow Edge with TrueV (Siemens Medical Solution, USA). A 30-sec infusion of 10 MBq/kg of Rubidium-82 chloride was administered intravenously using a Strontium-82/Rubidium-82 generator with a dedicated and automated software-controlled elution system (RubiFill, Jubilant Radiopharma, Kirkland, Canada) for both rest and stress imaging. Pharmacological stress was performed with dipyridamole or, in presence of dipyridamole contraindication, dobutamine. For patients aged 75 years or younger and without history of revascularization, calcium score was measured and reported when technically feasible with both SPECT and PET. For both modalities, ECG data were reported, and perfusion images were analyzed using Corridor4DM (INVIA Medical Imaging Solutions, Ann Arbor, Michigan, USA) by experienced physicians. Flow quantification was incorporated in the overall interpretation of PET MPI and reported.

### Statistical Analysis

The study objective was to compare performance outcomes of patients presenting with CP but no clear diagnosis of ACS at the ED when PET is available vs when PET is not available. In addition to the pre-defined outcomes, analyses were performed by grouping patients who underwent MPI based on modality (SPECT vs PET), regardless of the period. Epicardial disease on coronary angiography was considered significant in the presence of at least one of the following: luminal stenosis ≥70%, FFR ≤0.80, iFR <0.90, or stent implantation. LoS was defined as time interval between triage and ED discharge or hospital admission recorded in the ED database. Additional testing was defined as additional coronary computed tomography angiography (cCTA), treadmill stress test (TST), stress echocardiography, or MPI, performed within 3 months following the ED visit.

Categorical variables are presented as percent (%) and continuous variables as mean±SD with the exception of LoS which is presented as median (interquartile range, IQR). All statistical analyses were two-tailed and a *P* value of <0.05 was considered significant. All analyses are considered exploratory and no penalty for multiple testing has been applied. Categorical variables were compared using Fisher’s exact test when there were two categories and by Chi-square tests when there were more than two categories. Continuous variables were compared with Student’s two-sample *t* test. LoS between the different groups were analyzed with Kolmogorov–Smirnov tests to compare the cumulative distributions. Linear regressions were used to assess trends over time. Patients with missing or incomplete data were excluded from analyses. Statistical analyses were performed using GraphPad Prism version 9.0.0 for Windows.

## Results

**Figure 2:**
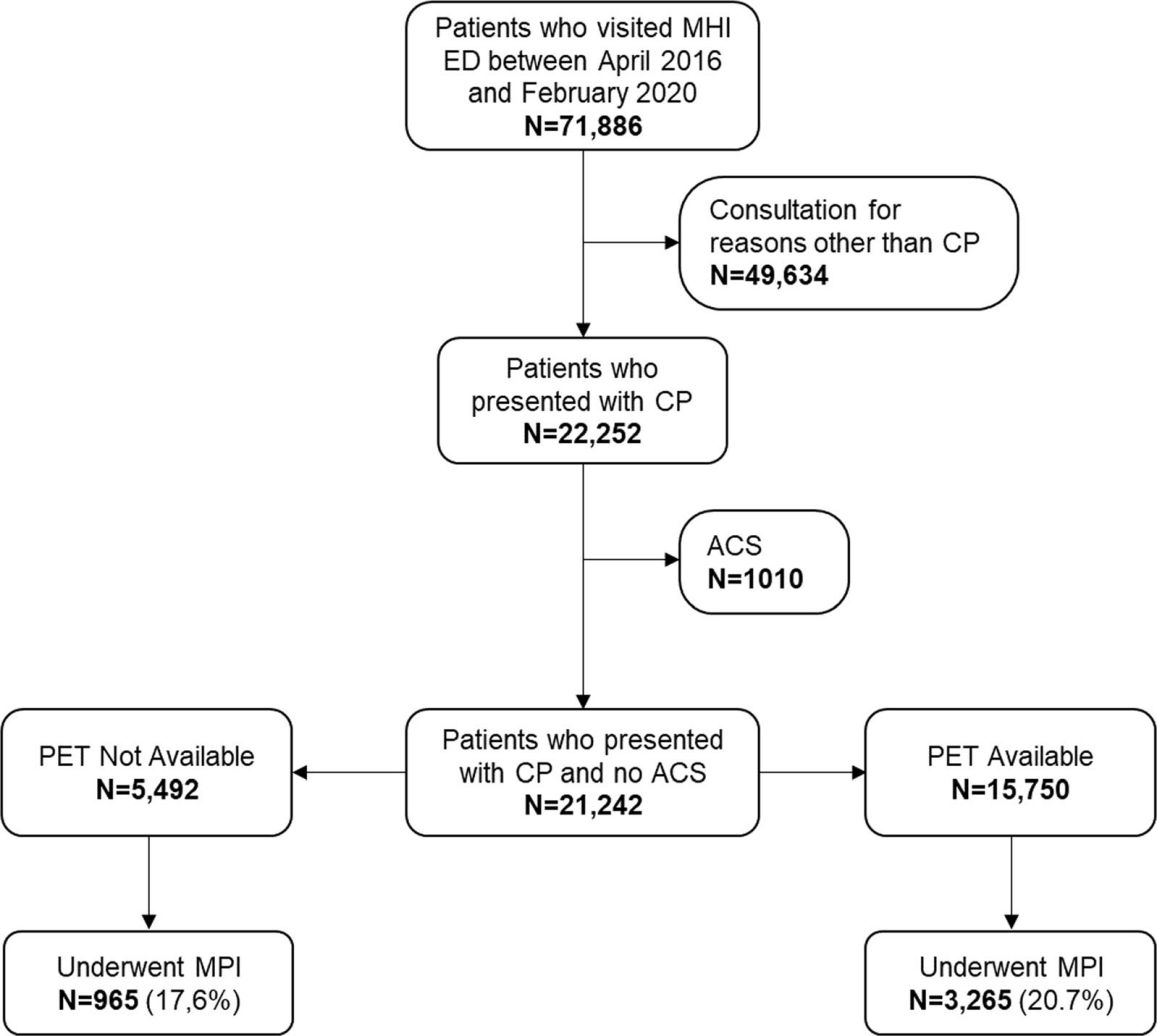
Study population flow chart

**Figure 3:**
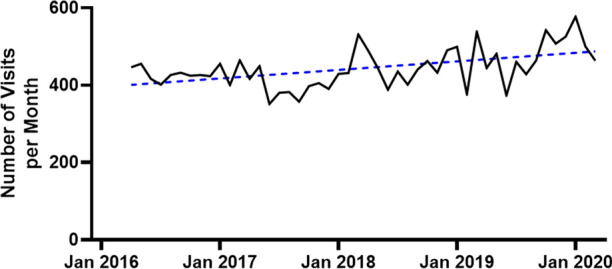
Number of ED visits for chest pain, excluding patients with definite ACS

There were 71,886 patient visits at the MHI ED during the study period. A total of 22,252 visits presented with the chief complain of CP, of which 1,010 were attributed to a definite ACS and therefore excluded from the analyses (Figure 2). Thus, 21,242 visits were included in the analyses: 5,492 in the PET Not Available group and 15,750 in the PET Available group. In the PET Available group, 2277 (69.7%) patients who underwent MPI were imaged with PET and 988 (30.3%) were imaged with SPECT. Patient characteristics of both groups are presented in Table 1. On average, there were 442.5 ± 50.8 visits for CP each month. There was a mild but statistically significant increased number of visits over time (linear regression slope: 1.767 patients/month, *P* = 0.0004, Figure 3).

**Table 1:** Study group patient characteristics

	PET not available (N = 5,492)	PET available (N = 15,750)	*P* value
Age	62 ± 16	61 ± 16	1.00
Sex female	41.8%	41.8%	1.00
Blood work^a^			
hs-cTnI	123 ± 835 ng/L	130 ± 965 ng/L	0.62
CK	425 ± 1120 U/L	460 ± 989 U/L	0.53
NT-proBNP	2494 ± 4030 ng/L	2412 ± 4353 ng/L	0.69
CAD risk factors^b^			
Hypertension	67.9%	65.0%	0.41
Smoking			0.29
Never	39.1%	44.0%	
Remote	49.0%	43.5%	
Active	11.9%	12.5%	
Diabetes	26.3%	31.7%	0.11
Dyslipidemia	67.6%	67.2%	0.94
CAD history^b^			
Prior ICA	45.9%	47.5%	0.53
MI	26.4%	28.0%	0.44
PCI	29.0%	30.2%	0.55
CABG	15.6%	17.6%	0.21

*CABG* coronary artery by-pass graft surgery, *CAD* coronary artery disease, *CK* creatine kinase, *hs-cTnI* high-sensitivity cardiac troponin-I, *MI* myocardial infarction, *NT-proBNP* N-terminal pro-brain natriuretic peptide, *PCI* percutaneous coronary intervention

^a^Maximal value recorded during ED visit

^b^Data of patients who underwent MPI study for Period 2-4

The number of patients presenting at the MHI ED with CP and referred for a MPI study has been increasing following the introduction of PET in January 2017 (linear regression slope: 0.5 patients/month, *P* = 0.017), while the total number of MPI was stable over time preceding the introduction of PET (linear regression slope: − 0.08 patients/month, *P* = 0.92, Fig. 1). The absolute number of MPI referral from ED per month is 21.1% greater when PET is available compared to when PET is unavailable (63.1 vs 52.1, *P* = 0.013). The proportion of patients who presented with CP at the MHI ED and underwent a MPI study is greater when PET is available compared to when PET is not available (20.7% vs 17.6%, *P* < 0.0001).

**Figure 4:**
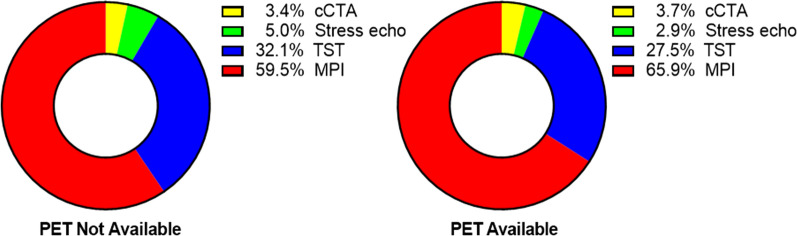
Proportion of non-invasive testing performed for patients presenting at the emergency department with chest pain during the periods where PET was not available (left) and PET was available (right). *cCTA* computed tomography angiography; *MPI* myocardial perfusion imaging, *TST* treadmill stress test

Among patients who underwent risk stratification testing, including cCTA, stress echo, TST, and MPI, MPI was chosen 65.9% of the time when PET was available compared to 59.5% when PET was not available (*P* < 0.0001, Figure 4).

### Coronary Angiography

The proportion of patients presenting with CP at the MHI ED undergoing coronary angiography was not different when PET was available compared to when PET was not available (14.3% vs 14.2%, *P* = 0.93). Among patients presenting with CP at the ED undergoing coronary angiography, the proportion of coronary angiography without significant CAD was similar when PET was available compared to when PET was not available (18.5% vs 21.4%, *P* = 0.24). The proportion of patients undergoing angioplasty was not different when PET was available vs not available (6.3% vs 6.4%, *P* = 0.86).

Among patients who underwent both an MPI study and a coronary angiogram, the proportion of coronary angiogram without significant epicardial CAD tended to be greater in those who underwent SPECT MPI compared to those who underwent PET MPI (21.2% vs 14.2% *P* = 0.09) regardless of the period, but the difference did not reach statistical significance.

### Length of Stay

**Figure 5:**
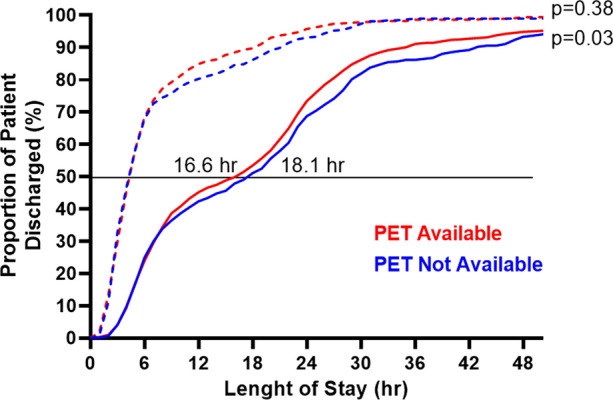
Cumulative distribution of discharge from ED according to the length of stay of patients presenting with CP and undergoing MPI imaging prior to discharge (bold lines) and after discharge (dotted lines). *PET* positron emission tomography

Median ED LoS of patients who underwent MPI risk stratification prior to discharge was shorter when PET was available 16.6 (IQR: 6.7-25.2) hours compared to 18.1 (IQR: 6.5-27.8) hours when PET was not available (*P* = 0.03) (Figure 5). Median LoS in the ED of patients who underwent subsequent elective MPI risk stratification as outpatients was not different when PET was available 4.8 (IQR: 3.2-7.8) hours compared to 4.8 (IQR: 3.1-8.9) hours when PET is not available (*P* = 0.38). Median ED LoS of patients who underwent MPI risk stratification prior to discharge was 17.7 (IQR: 6.5-28.2) hours, 16.6 (IQR: 6.6-24.6) hours, 18.7 (IQR: 6.5-24.3) hours, and 16.6 (IQR: 6.8-26.0) hours for Periods 1, 2, 3, and 4, respectively.

### Downstream Testing

Patients who underwent SPECT MPI had significantly more downstream testing (cCTA, stress echo, TST, repeated MPI) in the following 3 months compared to those who underwent PET MPI (8.9% vs 6.4%, *P* = 0.003).

## Discussion

This is the first large and comprehensive study to evaluate the impact and influence of PET MPI on the management of patients with CP in the ED. PET MPI presents several advantages over SPECT MPI, including improved diagnostic accuracy, favorable dosimetry, and ability to accurately quantify myocardial blood flow in absolute units of mL/min/g.^9,10^ Importantly, for best utilization of a nuclear cardiology department, a full rest-stress PET MPI study with Rubidium-82 can be completed within 30-45 minutes. These advantages, especially from an ED or chest pain unit perspective, present Rubidium-82 PET MPI as an attractive alternative to SPECT MPI. Despite the PET MPI advantages noted above, very few studies have evaluated PET MPI’s role in the ED setting. Osborne et al. reported that Rubidium-82 PET MPI and coronary cCTA have similar true-positive rates in patients with low-risk acute chest pain,^11^ although no studies have evaluated the real-world impact of Rubidium-82 PET on ED performance indicators or patient management. In this study, we observed that availability of PET MPI is associated with an increased number of MPI referrals from the ED coupled with a decreased LoS for patients imaged prior to discharge and a significant reduction in downstream testing. Curiously, these benefits were not associated with a significantly lower rate of coronary angiography exams without significant epicardial stenoses.

The introduction of PET MPI at the MHI led to significant changes in the management of patients presenting with CP but without definite ACS. During the study period, there has been an increase in the number of MPI studies requested by ED physicians, outpacing the increase in ED visits for CP. This translated in an increase proportion of patients undergoing MPI studies, PET, or SPECT, over time. This increase in proportion of patients undergoing MPI studies resulted in a decreased proportion of patients undergoing TST and, to a lesser extent, a decreased proportion of patients undergoing stress echocardiography. Several factors could account for this referral shift, although this study does not specifically address this question. Such factors include improved efficiency of MPI imaging with the introduction of PET, fewer equivocal or non-diagnostic studies with PET MPI compared to SPECT MPI, increased confidence of the referring physician, as well as improved patient throughput with PET leading to increased test availability. Although the proportion of patients undergoing coronary cCTA was essentially unchanged, this might be explained by the low proportion of patients undergoing cCTA. Another explanation or contributor to this observation is the fact that patients presenting with CP at our institution are often at higher risk of CAD and/or have a history of revascularization.

Availability of PET MPI was associated with shorter LoS in ED for patients who underwent imaging prior to discharge, with 50% of patients discharge after 16.6 hours compared to 18.1 hours when PET is not available. This 90-minute decrease of median LoS can be explained, at least in part, by the fact that imaging with Rubidium-82 is significantly shorter compared to SPECT MPI. Remarkably, a decrease in LoS was observed despite an increase in ED chest pain visits as well as an increase in the number of MPI performed when PET is available. Unsurprisingly, LoS for those imaged as outpatient was not different, as time to decision should not be affected by imaging duration. Several factors must be taken into consideration with regards to the reported LoS. The MHI ED operates like a chest pain unit as some patients may be observed for several hours to monitor ECG and serum cardiac markers prior to imaging, which may account for the relatively long LoS. In addition, is important to note that at our institution, SPECT MPI has been widely used for several years in the evaluation of ED patients with CP. Indeed, prior to the introduction of PET MPI, our center performed on average approximately two (2) SPECT MPI studies daily for patients with CP presenting at the ED. The actual number of patients referred from the ED for a MPI study is even greater when other indications such as dyspnea and arrhythmia are considered. In that context, the logistics of SPECT MPI for ED referrals have already been optimized for several years. This is supported by the relatively low median LoS in the ED for patients undergoing SPECT MPI (18.1 hours) when compared to standard of care in the ROMICAT-II trial (26.7 hours). Thus, introduction of PET MPI could potentially lead to a more substantial increase in referrals and decreased LoS in centers where SPECT MPI is not already routinely performed and optimized for ED or chest pain unit patients. Other possible contributing factors to this difference are higher accuracy, less downstream testing, and potentially fewer equivocal studies with PET MPI compared to SPECT MPI.

An interesting finding of this study is the fact that patients who underwent PET MPI had − 40% less chances of having additional testing within 3 months compared to those who underwent SPECT MPI. Fewer equivocal or non-diagnostic studies with PET MPI compared to SPECT MPI may contribute to this difference. A critical PET advantage is its ability to provide absolute quantification of myocardial blood flow and myocardial flow reserve which provides incremental diagnostic and prognostic information that may alleviate the need for further testing. Importantly, myocardial blood flow and flow reserve were reported and integrated in the final interpretation of the PET MPI studies, which may have contributed to this finding. Finally, increased confidence of the referring physicians in test results may also contribute to fewer supplemental tests.

In our study, the introduction of PET did not reduce the proportion of patients undergoing coronary angiography. Nonetheless, the proportion of coronary angiography without significant epicardial CAD was slightly lower (18.5% vs 21.4%) when PET was available compared to when PET was not available. Similarly, the proportion of coronary angiography without significant epicardial disease was also lower following a PET MPI vs a SPECT MPI (14.2% vs 21.2%). Although the differences did not reach statistical significance, this trend is not surprising given the superior diagnostic accuracy of PET MPI compared to SPECT MPI. It is reasonable to believe that these numbers may further decrease as the referring ED physicians and clinicians gain more experience and trust in the PET findings for ED patients. In addition, it is important to note that state-of-the-art SPECT/CT imaging with cardiofocal collimators and attenuation correction was systematically used when for SPECT MPI, potentially attenuating the differences observed between PET and SPECT. This suggests that although the findings in this study may appear on the surface to be relatively modest, when compared versus standards of care currently being used in the community, data in this paper are critically important in potentially improving the care of acute chest pain center patients. Furthermore, these results must take into consideration several factors. First, even when PET is available, some patients may be imaged with SPECT when the number of ED requisitions exceeds PET capacity. In these circumstances, SPECT is reserved for low-risk patients with BMI<30 kg/m^2^ and without history of CAD. This approach introduces a specificity bias when comparing rates of normal coronary angiogram following PET and SPECT. Similarly, rates of abnormal coronary angiography must not be confused with test specificity as patients may be referred for invasive testing for other valid clinical questions regardless of their negative/normal MPI results. It is interesting to note that in the ROMICAT-II trial,^12^ which was a randomized controlled study comparing cCTA vs standard of care evaluation for patients presenting with acute CP, utilization of cCTA yielded significantly shorter median LoS (8.6 hours) but at the cost of increased downstream testing, coronary angiography, and revascularization. Importantly, in this study, PET availability was not associated with an increase in angiography or angioplasty along with a reduction in downstream testing. Finally, another consideration is that patients are sometimes referred for a PET MPI to assess the hemodynamic significance of a known intermediate epicardial lesion or to localize ischemia prior a scheduled ICA, which can contribute to increase the proportion of patients who will undergo ICA following PET MPI.

## Limitations

There are unavoidable limitations in this single-center study from a quaternary cardiovascular medical center. These limitations must be taken into consideration when extrapolating these findings to other institutions, including assessing the generalizability and applicability of the results from a speciality referral center. Since this is a retrospective study, uncontrolled confounders or bias may contribute to the findings as well as the duration of the data collection; specifically, there was no correction for the learning curve of referring physician’s trust in the superior cardiac PET evaluation performance. It is also likely that clinicians were biased in favor of PET, which may have contributed to the differences in downstream testing observed between PET and SPECT. It is provocative and worth noting that the study periods when PET was unavailable (Period 1 and 3) were alternating with periods during which PET was available (Period 2 and 4), which may lessen the bias of confounders unrelated to imaging technique by partially emulating a natural randomization to either imaging strategy. However, this is still limited by the fact that seasonal variations in ED attendance are not accounted for. Nonetheless, the unique results of this study represent the actual impact of the introduction of PET MPI in a real-world clinical setting in an ED specializing in chest and cardiac evaluation and triage. Therefore, the results of this study do relate to the effectiveness of PET MPI, reflecting its true impact on the actual clinical practice. Said another way, a differently conducted trial might not present the advantages shown in a real-world application versus potential artificial, and perhaps not reproducible, efficacy from a highly controlled research environment.

Finally, in this study, PET MPI was exclusively performed with Rubidium-82. Applying these results to other PET MPI tracers, such as ^13^N-ammonia and ^18^F-flurpiridaz, although tempting and possibly true, is not straightforward and would require validation. Indeed, despite their diagnostic accuracy being comparable, other important differences must be taken into account.^9^ Foremost, Rubidium-82 is generator produced. It is therefore readily available for injection whenever needed, giving it a logistical advantage over cyclotron-produced tracers. As well, Rubidium-82 has a very short half-life of 75 seconds which allows rapid rest-stress imaging within 30-45 minutes.

## New Knowledge Gained

Availability of PET-MPI was associated with an increased number of MPI referrals from the ED for the evaluation of patients with acute CP as well as shorter ED LoS. In addition, utilization of PET MPI was associated with 40% fewer downstream testing compared to SPECT MPI in patients evaluated with acute CP at the ED.

## Conclusion

We observed that availability of PET MPI is associated with an increased number of MPI referral from the ED for the evaluation of patients with CP. In our cohort, PET availability was not associated with a statistically significant lower rate of coronary angiography without significant epicardial stenoses. Among patients undergoing non-invasive testing for evaluation of CP, MPI is the modality most frequently chosen by the referring physician when PET is available. In addition, PET MPI availability is also associated with a decreased ED LoS for patients imaged prior to discharge. Finally, patients undergoing PET MPI have significantly fewer downstream testing compared to those undergoing SPECT MPI.

## Supplementary Information

Below is the link to the electronic supplementary material.

Supplementary file1 (PPTX 5020 kb)

## Abbreviations

ACS: Acute coronary syndrome
AUC: Appropriate use criteria
BMI: Body mass index
CP: Chest pain
cCTA: Coronary computed tomography angiography
FFR: Fractional flow reserve
ED: Emergency department
iFR: Instantaneous wave-free ratio
IQR: Interquartile range
LoS: Length of stay
MHI: Montreal Heart Institute
MPI: Myocardial perfusion imaging
NSTEMI: Non-ST-elevation myocardial infarction
PET: Positron emission tomography
SPECT: Single photon emission computed tomography
STEMI: ST-elevation myocardial infarction
TST: Treadmill stress test
