# Supplementary material for: Impact of rubidium imaging availability on management of patients with acute chest pain
Source: J Nucl Cardiol. 2022 Feb 23;29(6):3281–90. doi: 10.1007/s12350-022-02923-8 (PMC8865882; doi:10.1007/s12350-022-02923-8)
Supplement: Supplementary file 1 — Supplementary file1 (PPTX 5020 kb) [file 12350_2022_2923_MOESM1_ESM.pptx]

## Slide 1
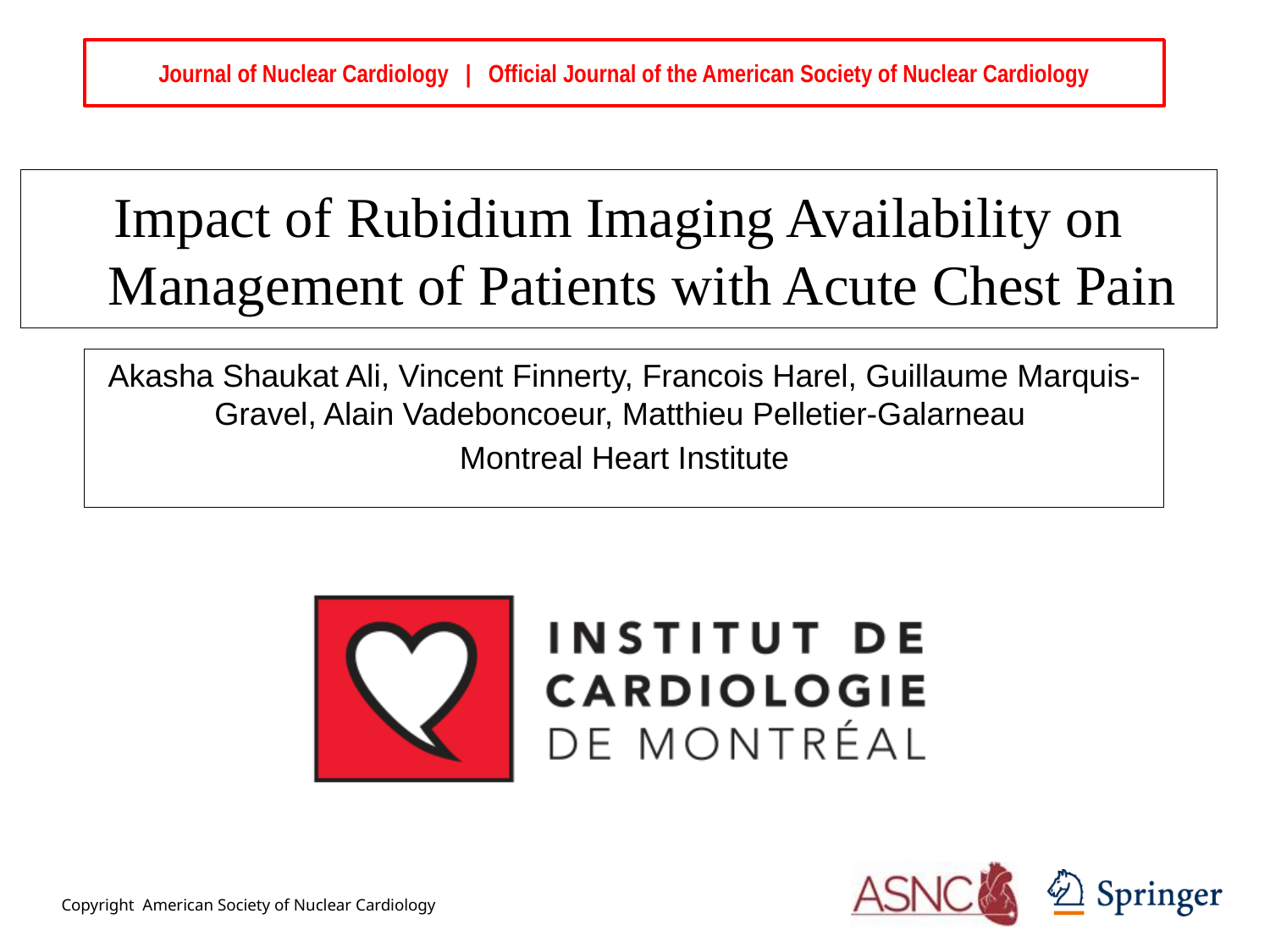

Journal of Nuclear Cardiology | Official Journal of the American Society of Nuclear Cardiology
# Impact of Rubidium Imaging Availability on Management of Patients with Acute Chest Pain
Akasha Shaukat Ali, Vincent Finnerty, Francois Harel, Guillaume Marquis-Gravel, Alain Vadeboncoeur, Matthieu Pelletier-Galarneau
Montreal Heart Institute
Copyright American Society of Nuclear Cardiology

## Slide 2
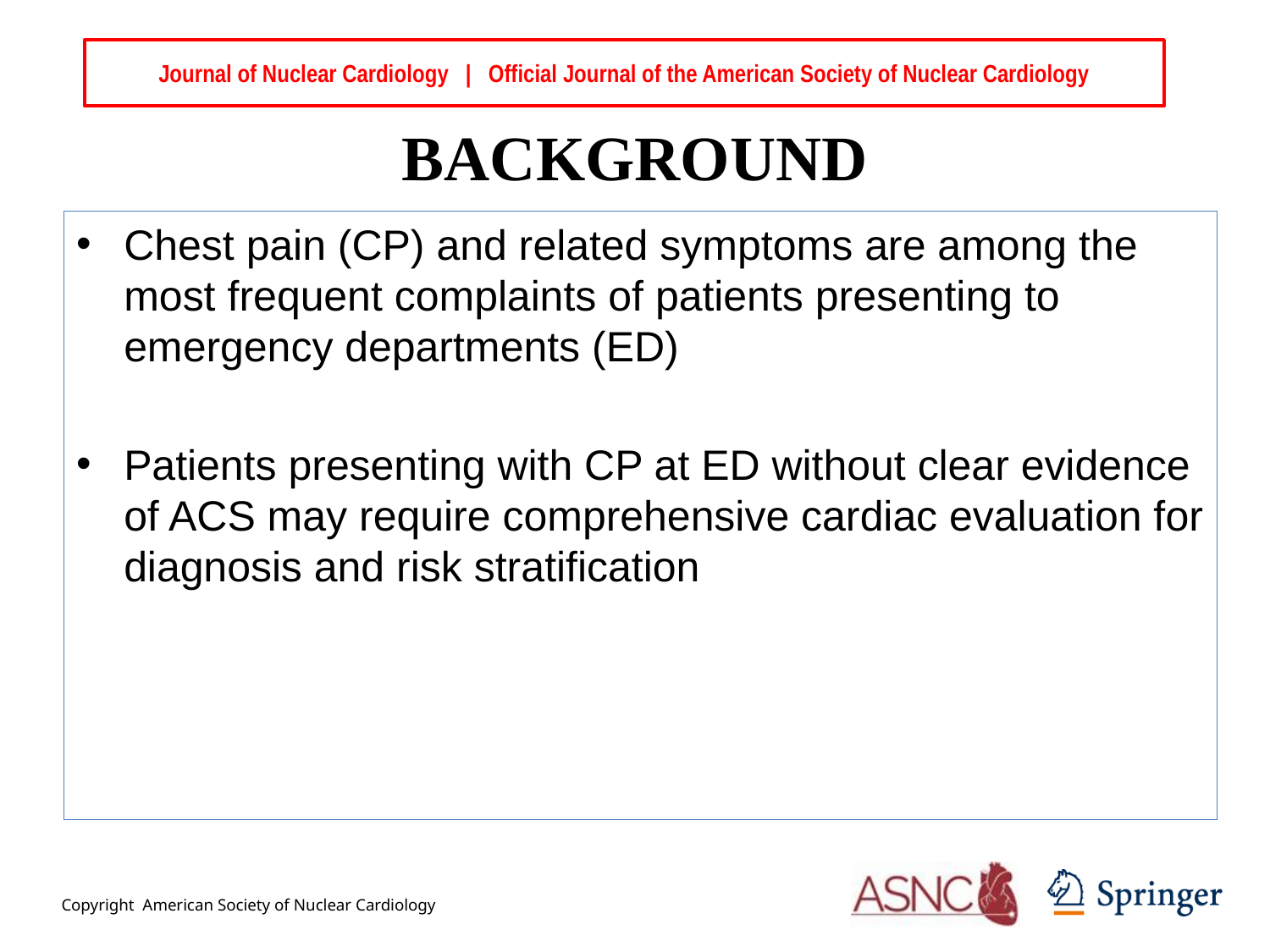

Journal of Nuclear Cardiology | Official Journal of the American Society of Nuclear Cardiology
# BACKGROUND
Chest pain (CP) and related symptoms are among the most frequent complaints of patients presenting to emergency departments (ED)
Patients presenting with CP at ED without clear evidence of ACS may require comprehensive cardiac evaluation for diagnosis and risk stratification
Copyright American Society of Nuclear Cardiology

## Slide 3
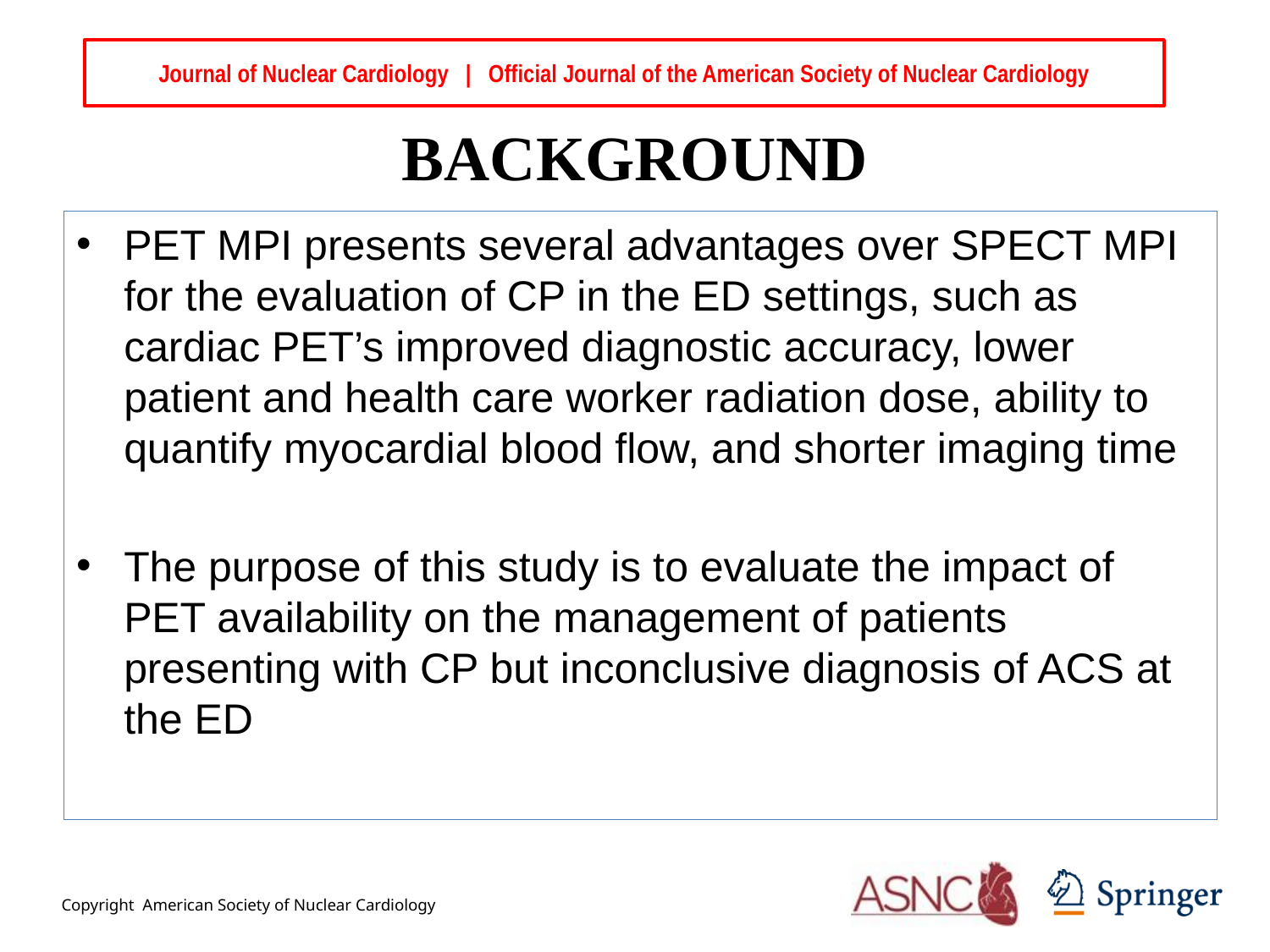

Journal of Nuclear Cardiology | Official Journal of the American Society of Nuclear Cardiology
# BACKGROUND
PET MPI presents several advantages over SPECT MPI for the evaluation of CP in the ED settings, such as cardiac PET’s improved diagnostic accuracy, lower patient and health care worker radiation dose, ability to quantify myocardial blood flow, and shorter imaging time
The purpose of this study is to evaluate the impact of PET availability on the management of patients presenting with CP but inconclusive diagnosis of ACS at the ED
Copyright American Society of Nuclear Cardiology

## Slide 4
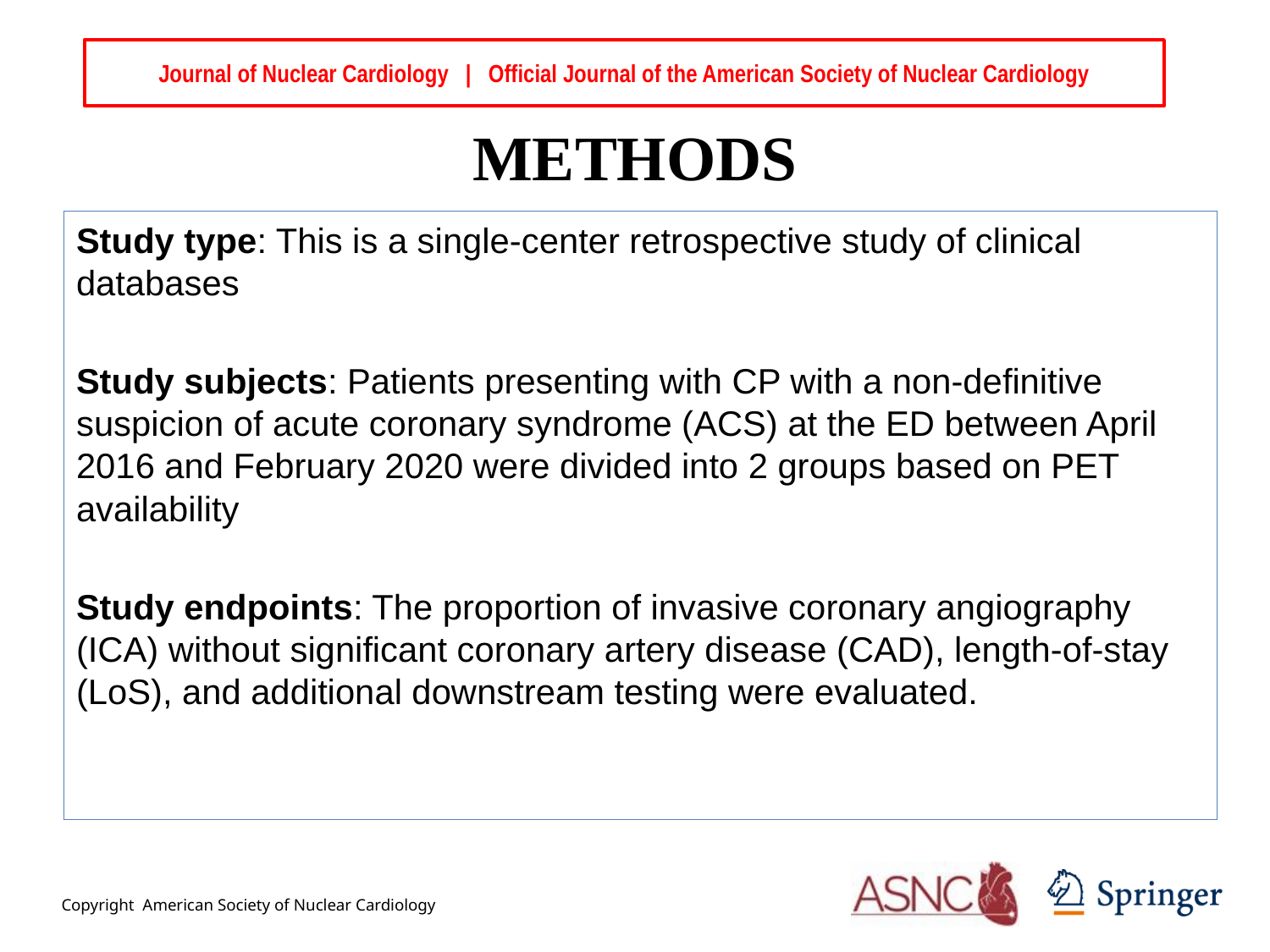

Journal of Nuclear Cardiology | Official Journal of the American Society of Nuclear Cardiology
# METHODS
Study type: This is a single-center retrospective study of clinical databases
Study subjects: Patients presenting with CP with a non-definitive suspicion of acute coronary syndrome (ACS) at the ED between April 2016 and February 2020 were divided into 2 groups based on PET availability
Study endpoints: The proportion of invasive coronary angiography (ICA) without significant coronary artery disease (CAD), length-of-stay (LoS), and additional downstream testing were evaluated.
Copyright American Society of Nuclear Cardiology

## Slide 5
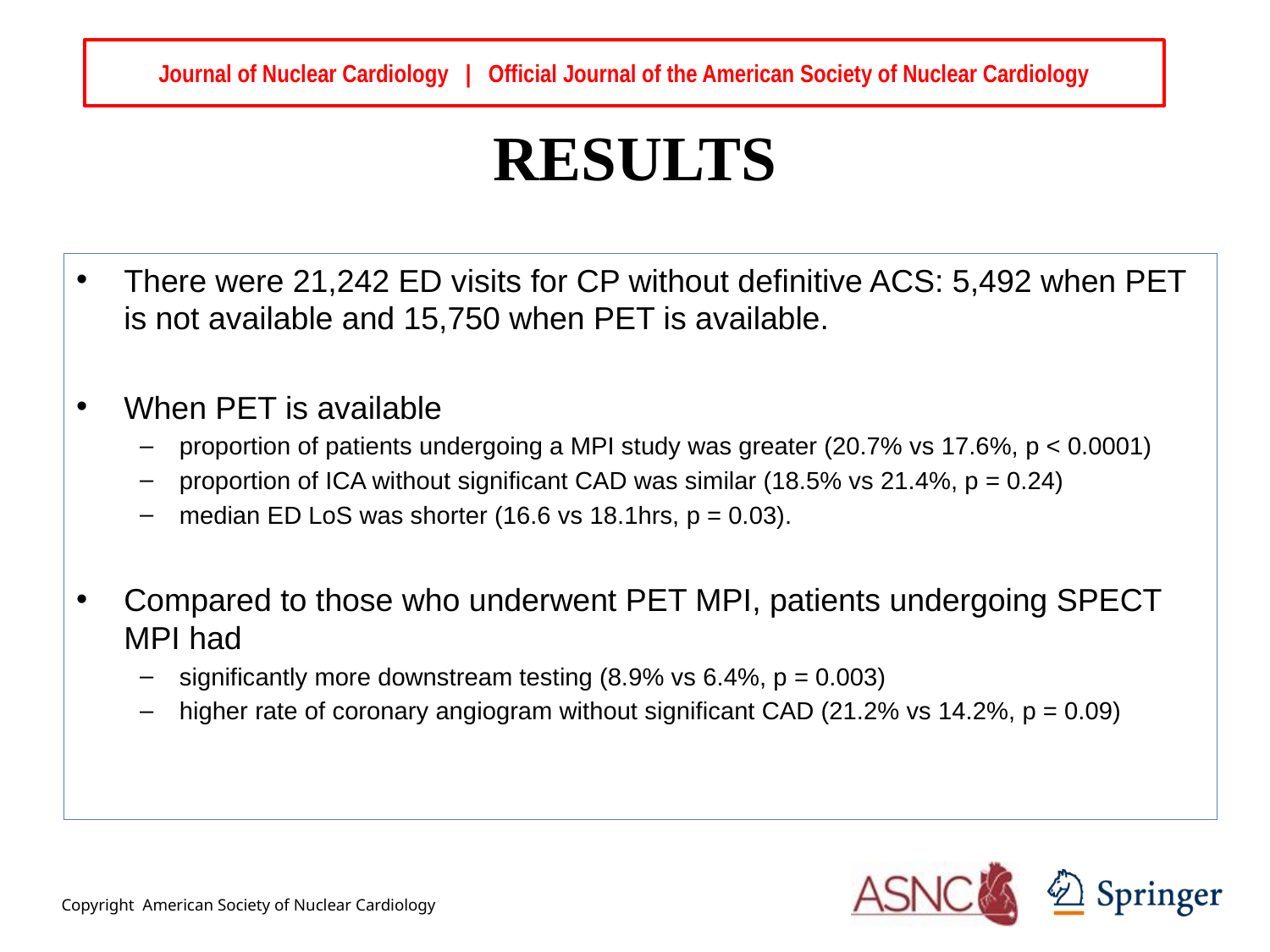

Journal of Nuclear Cardiology | Official Journal of the American Society of Nuclear Cardiology
# RESULTS
There were 21,242 ED visits for CP without definitive ACS: 5,492 when PET is not available and 15,750 when PET is available.
When PET is available
proportion of patients undergoing a MPI study was greater (20.7% vs 17.6%, p < 0.0001)
proportion of ICA without significant CAD was similar (18.5% vs 21.4%, p = 0.24)
median ED LoS was shorter (16.6 vs 18.1hrs, p = 0.03).
Compared to those who underwent PET MPI, patients undergoing SPECT MPI had
significantly more downstream testing (8.9% vs 6.4%, p = 0.003)
higher rate of coronary angiogram without significant CAD (21.2% vs 14.2%, p = 0.09)
Copyright American Society of Nuclear Cardiology

## Slide 6
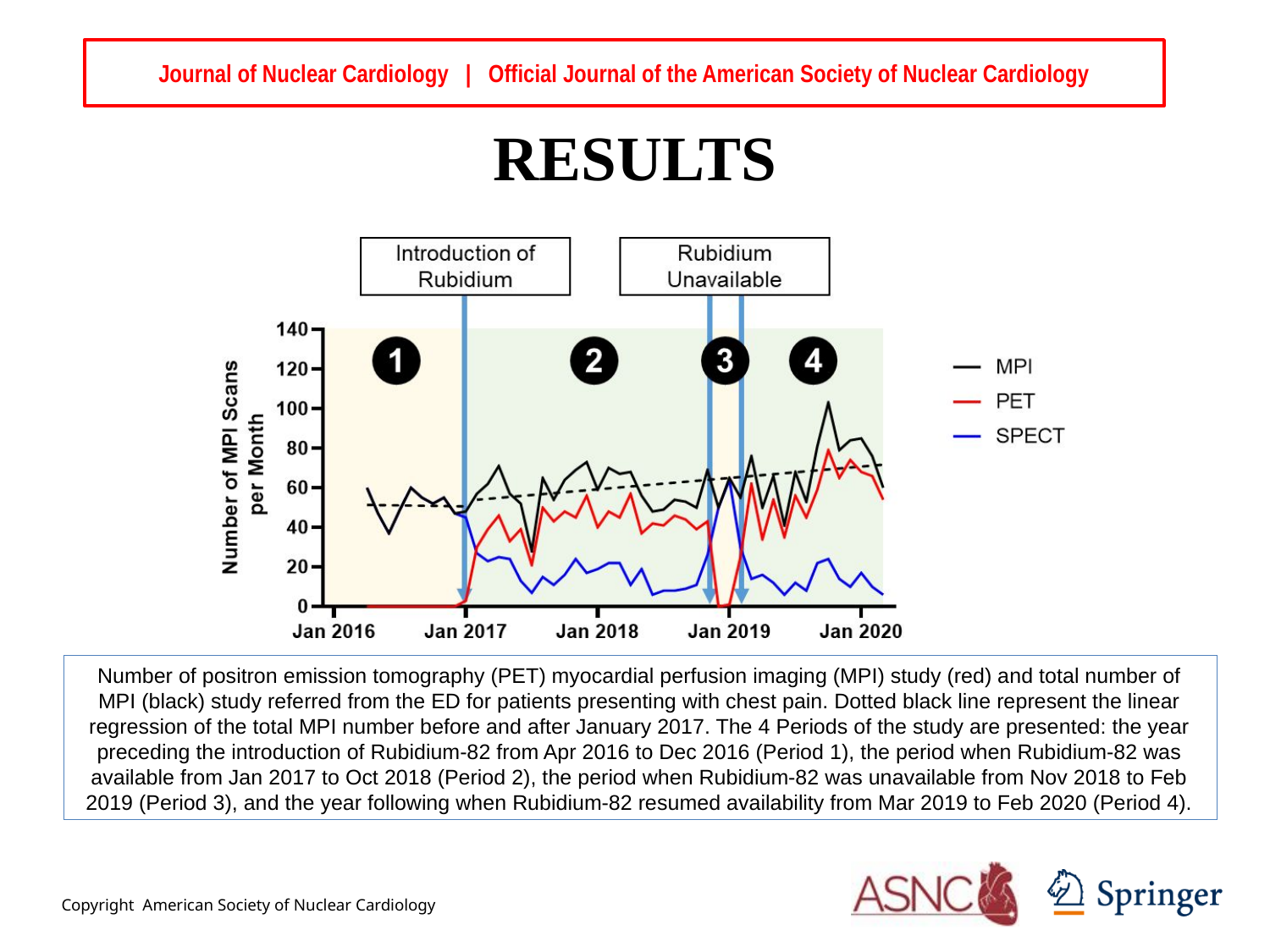

Journal of Nuclear Cardiology | Official Journal of the American Society of Nuclear Cardiology
# RESULTS
Number of positron emission tomography (PET) myocardial perfusion imaging (MPI) study (red) and total number of MPI (black) study referred from the ED for patients presenting with chest pain. Dotted black line represent the linear regression of the total MPI number before and after January 2017. The 4 Periods of the study are presented: the year preceding the introduction of Rubidium-82 from Apr 2016 to Dec 2016 (Period 1), the period when Rubidium-82 was available from Jan 2017 to Oct 2018 (Period 2), the period when Rubidium-82 was unavailable from Nov 2018 to Feb 2019 (Period 3), and the year following when Rubidium-82 resumed availability from Mar 2019 to Feb 2020 (Period 4).
Copyright American Society of Nuclear Cardiology

## Slide 7
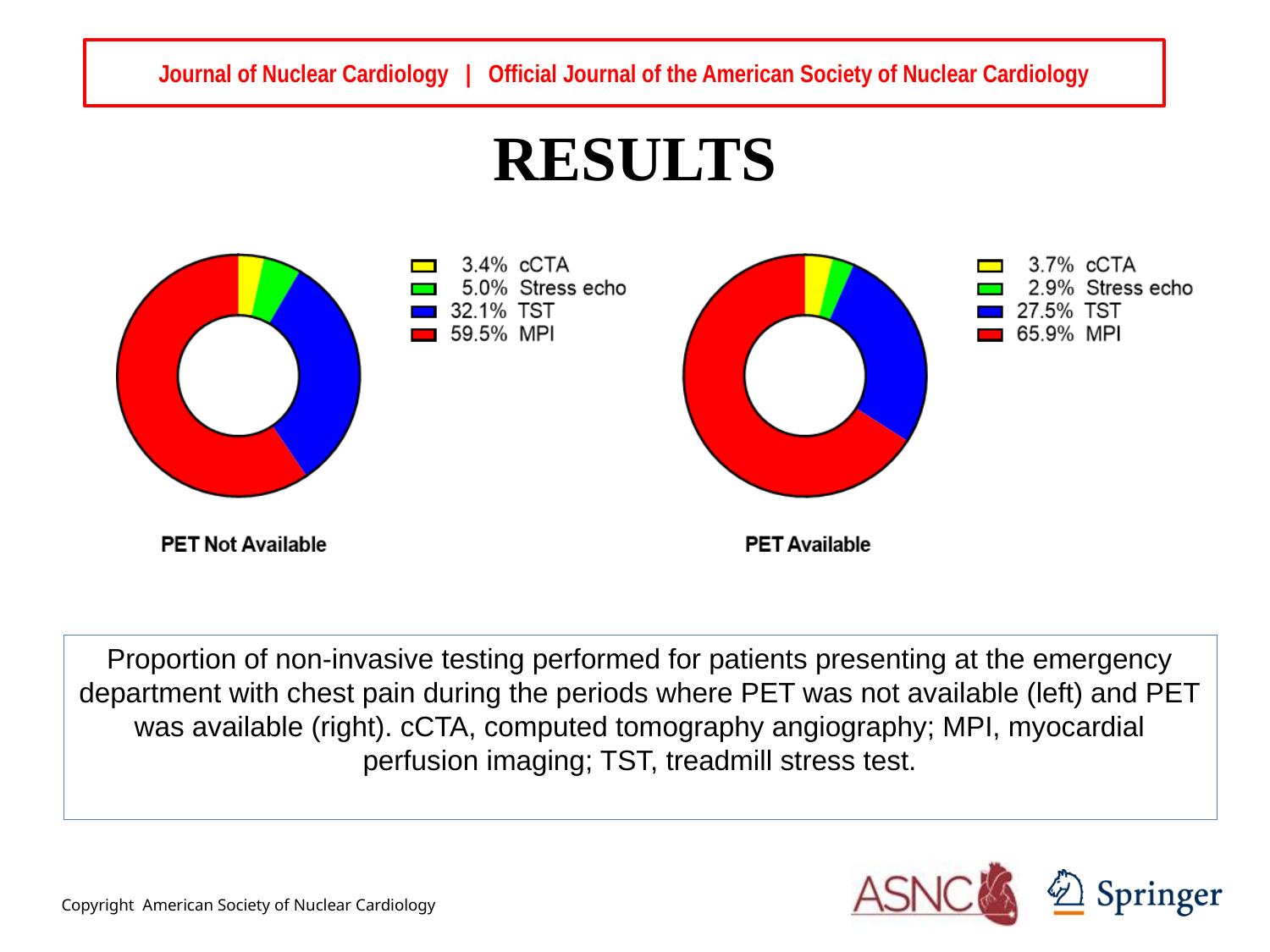

Journal of Nuclear Cardiology | Official Journal of the American Society of Nuclear Cardiology
# RESULTS
Proportion of non-invasive testing performed for patients presenting at the emergency department with chest pain during the periods where PET was not available (left) and PET was available (right). cCTA, computed tomography angiography; MPI, myocardial perfusion imaging; TST, treadmill stress test.
Copyright American Society of Nuclear Cardiology

## Slide 8
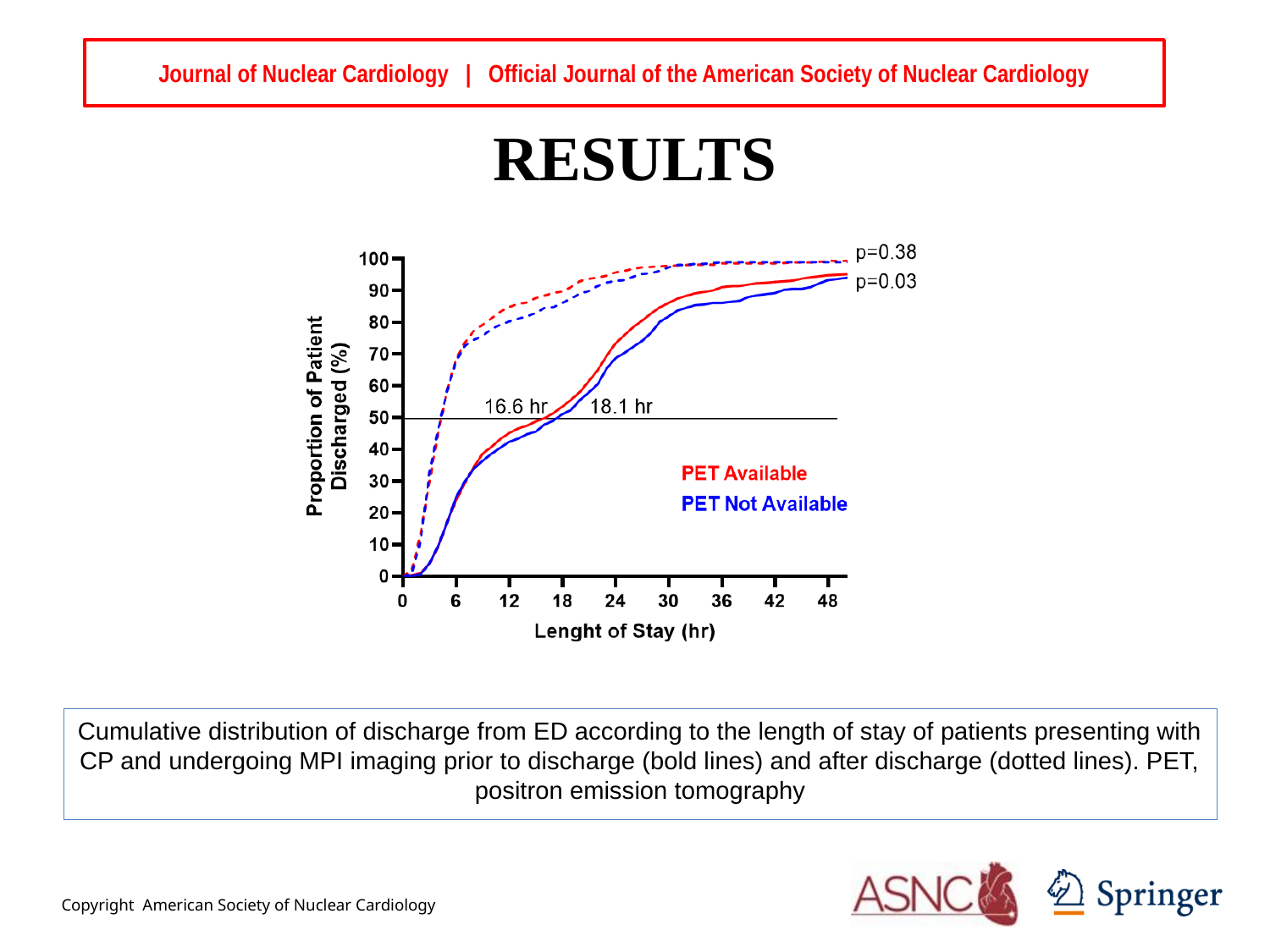

Journal of Nuclear Cardiology | Official Journal of the American Society of Nuclear Cardiology
# RESULTS
Cumulative distribution of discharge from ED according to the length of stay of patients presenting with CP and undergoing MPI imaging prior to discharge (bold lines) and after discharge (dotted lines). PET, positron emission tomography
Copyright American Society of Nuclear Cardiology

## Slide 9
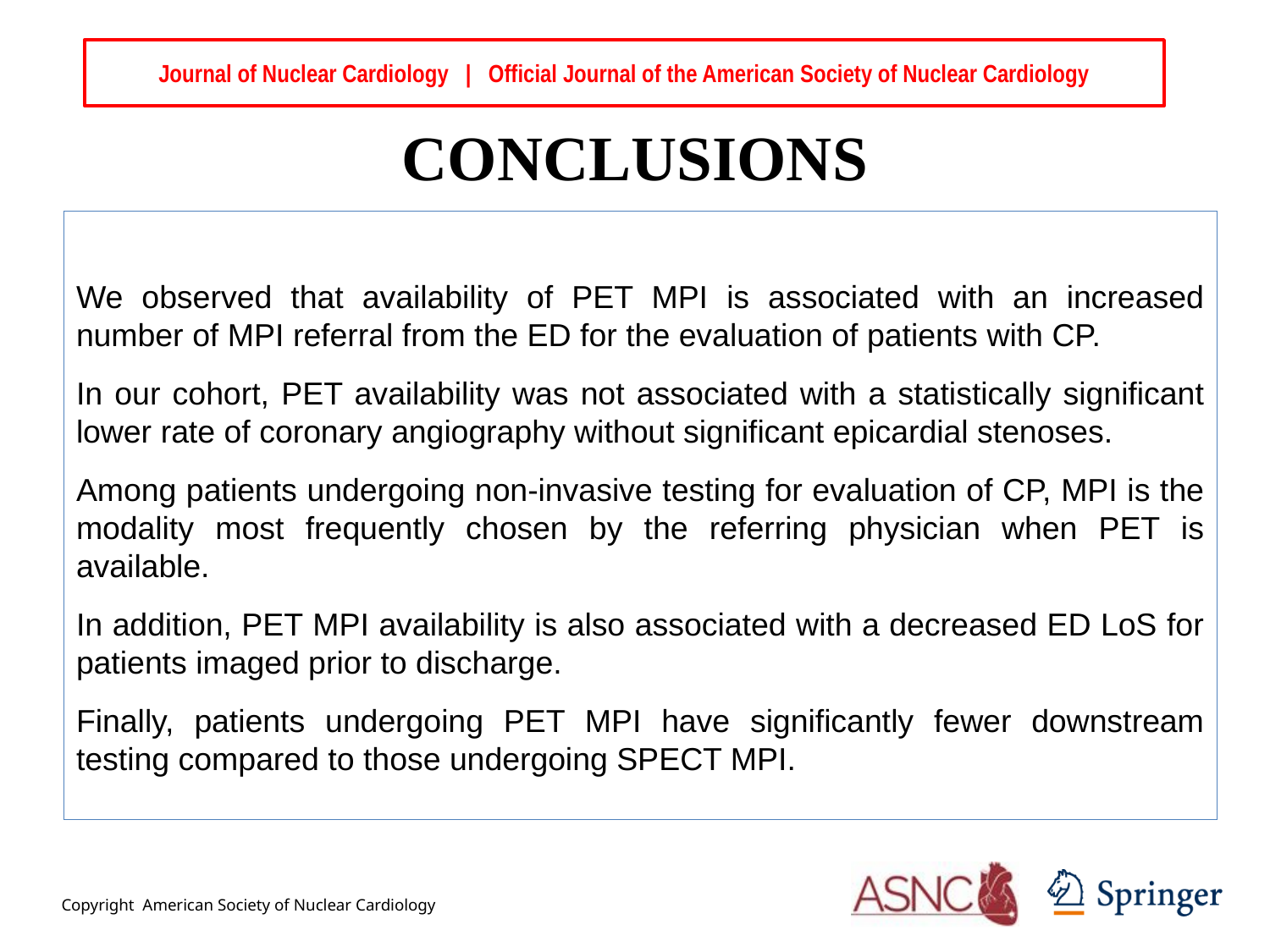

Journal of Nuclear Cardiology | Official Journal of the American Society of Nuclear Cardiology
# CONCLUSIONS
We observed that availability of PET MPI is associated with an increased number of MPI referral from the ED for the evaluation of patients with CP.
In our cohort, PET availability was not associated with a statistically significant lower rate of coronary angiography without significant epicardial stenoses.
Among patients undergoing non-invasive testing for evaluation of CP, MPI is the modality most frequently chosen by the referring physician when PET is available.
In addition, PET MPI availability is also associated with a decreased ED LoS for patients imaged prior to discharge.
Finally, patients undergoing PET MPI have significantly fewer downstream testing compared to those undergoing SPECT MPI.
Copyright American Society of Nuclear Cardiology
